# The burden of COVID-19 death for different cancer types: a large population-based study

**DOI:** 10.7189/jogh.15.04046

**Published:** 2025-02-14

**Authors:** You Mo, Duncan Wei, Xiaozheng Chen, Zengfu Zhang, Wen Huo, Meng Wu, Dawei Chen, Jinming Yu

**Affiliations:** 1 Laboratory of Molecular Cardiology, The First Affiliated Hospital of Shantou University Medical College, Shantou, China; 2 Shandong Provincial Key Laboratory of Precision Oncology, Shandong Cancer Hospital and Institute, Shandong First Medical University and Shandong Academy of Medical Sciences, Jinan, China; 3 Department of Radiation Oncology, Affiliated Tumour Hospital of Xinjiang Medical University, Urumqi, China

## Abstract

**Background:**

Viral mutations and immune dysfunction still lead to recurrent infections of COVID-19 in cancer patients. Our aim in this study was to explore the differences in cumulative risk of COVID-19 death from different cancer types and characterise clinical and demographic factors associated with COVID-19 death.

**Methods:**

We conducted a population-based study using the National Cancer Database, which included all cancer types. We calculated age-standardised mortality, cancer mortality, and COVID-19 mortality. Further, we employed a multivariate competing risk analysis to calculate the cumulative risk of COVID-19 death in different cancer types.

**Results:**

5.3% of cancer patients suffered from COVID-19 death. The highest COVID-19 mortality was in chronic lymphocytic leukaemia, while lung and bronchus cancer exhibited lower risk. Notably, years from cancer diagnosis independently predict COVID-19 death. The hazard ratios (HR) in different types of cancers were as follows: lung and bronchus cancer HR = 1.29 (95% confidence interval (CI) = 1.20–1.40, *P* < 0.001), colon and rectum cancer HR = 1.22 (95% CI = 1.16–1.29, *P* < 0.001), urinary bladder cancer HR = 1.22 (95% CI = 1.15–1.30, *P* < 0.001), non-Hodgkin lymphoma HR = 1.17 (95% CI = 1.11–1.23, *P* < 0.001), kidney cancer HR = 1.15 (95% CI = 1.06–1.24, *P* < 0.001), and breast cancer HR = 1.11 (95% CI = 1.06–1.16, *P* < 0.001). Radiotherapy, chemotherapy, and surgical resection did not significantly correlate with COVID-19 death.

**Conclusions:**

We revealed the burden of COVID-19 death across different cancer types. COVID-19 mortality was highest in chronic lymphocytic leukaemia and prostate cancer, while patients with lung and bronchus cancer had a lower risk. Years from diagnosis independently predict COVID-19 death. Based on the results, we support more prompt risk assessment and treatment for various types of cancer.

The severe acute respiratory syndrome coronavirus 2 (SARS-CoV-2) pandemic has resulted in over 777 million confirmed cases and over 7.1 million deaths worldwide, as reported by the World Health Organization (WHO) [1]. Recurrent infections and long-term effects are endangering global health, particularly for vulnerable populations such as cancer patients [2–4]. Cancer is a major determinant of disease burden worldwide. Cancer patients have an increased risk for SARS-CoV-2 infection and worse outcomes compared to the general population [5–7]. On 11 March 2020, the WHO declared the COVID-19 pandemic a global health crisis. Between December 2020 and March 2023, COVID-19 vaccines reduced deaths by 59% compared to the COVID-19 pandemic 2020. Approximately 60% of lives were saved during the Omicron period (from December 2021 to January 2022) [8]. However, the viral mutations and immune dysfunction still lead to recurrent infections of COVID-19 in cancer patients [9,10]. Several factors contribute to the vulnerability of cancer patients. First, cancer patients tend to be older and have comorbidities, which make them more susceptible to severe illness of COVID-19. Additionally, cancer treatments and immunosuppressed conditions heighten risk [11]. Previous studies have shown that the 30-day mortality for COVID-19 in cancer patients ranges from 13–33% [12–14], compared with 0.1–2% in the general population [15]. These findings suggest that cancer is a risk factor for severe COVID-19. Therefore, health care providers need to take extra precautions to minimise the risk of SARS-CoV-2 infection and death.

Prior studies on the relationship between cancer and COVID-19 were limited by lower statistical power, particularly in the overall cancer population. Most studies have focused on cancer patients who were hospitalised with COVID-19. About 80% of patients enrolled in previous studies had solid tumours, with 292 out of the 4966 patients requiring mechanical ventilation, and 695 dying within 30 days [15]. Two systematic reviews have reported that patients with lung cancer and haematological malignancies have a higher mortality of COVID-19 [16,17]. Additionally, another report showed that 19.6% of COVID-19 infection cases are with lung cancer, 18.7% with gastrointestinal, and 18.7% with genitourinary cancers [18] However, these studies enrolled only patients who needed to be hospitalised, and they could not represent the total population of cancer patients.

Despite several studies exploring the relationship between cancer types and COVID-19 outcomes, significant heterogeneity remains in cancer types. Chronic lymphocytic leukaemia (CLL) treatment may increase susceptibility to severe COVID-19 due to immune dysfunction [19]. Patients with lung cancer exhibited a significantly increased mortality risk and severity than other cancer types [16,20,21]. The results of some studies are inconsistent, such as the protective effect of anti-leukaemia therapy on CLL patients infected with COVID-19 and the low risk of serious events in lung cancer patients [5,21,22]. This can be partly explained by the heterogeneous population with cancers in different studies. A more comprehensive understanding of the risk factors for COVID-19 death across various cancer types is eagerly needed.

Limited data are currently available on COVID-19 death among cancer patients based on a large-scale cohort. Thus, we aimed to explore the differences in cumulative risk of COVID-19 death among various cancer types. We also characterised clinical and demographic factors to identify predictors of COVID-19 death in cancer patients.

## METHODS

### Setting and data sources

In conducting this study, we followed the Strengthening the Reporting of Observational Studies in Epidemiology (STROBE) guidelines for observational studies. We chose the study population from the Surveillance, Epidemiology, and End Results (SEER) database based on the November 2022 submission covering the period between 2000–20 (17 registries, November 2022), which was updated in April 2023. We calculated age-standardised mortality, cancer mortality, and COVID-19 mortality.

### Data collection

This paper contains an analysis of the April 2023 SEER registry data submission to the National Cancer Institute. We utilised the SEER*Stat, version 8.4.1 (National Cancer Institute, Bethesda, MD, USA) to extract data, which included patient demographics, clinicopathological features, treatment, and follow-up information for vital status. 285 708 patients were enrolled for our current study (patient population died in 2020), using cause of death to site recode based on International Classification of Diseases for Oncology (3rd edition). We considered patients to have died of COVID-19 infection when cause of death recode in SEER database was 103. This corresponds with the following International Statistical Classification of Diseases and Related Health Problems 10th revision code I071. We found 10 456 patients to be missing survival months and vital status. Due to the presence of 37 896 patients with repeated follow-ups, we selected only the first matching record for each person. Consequently, we enrolled the 237 356 patients in this study (Figure S1 in the **Online Supplementary Document**).

### Data analysis

We performed the statistical analyses and cartography using Stata, version 16 (College Station, TX, USA), Graph Pad Prism, version 8.0 (GraphPad Software, San Diego, CA, USA), and *R*, version 4.2.3 (R Core Team, Vienna, Austria). We employed a joinpoint segmented regression to analyse the changing trends of cancer incidence and mortality over time and to detect if there are points of trend change [22]. This method helps understand the dynamic changes in disease development, providing important insights for formulating prevention and intervention strategies. We established piecewise regression to characterise time trends in mortality. We calculated the annual percentage change (APC) for the age-adjusted mortality rates using the Joinpoint Regression Program, version 5.3.0 (National Cancer Institute, Bethesda, MD, USA) [23]. An asterisk indicates that the APC significantly differs from zero (*P* < 0.05). We expressed continuous variables as means and standard deviation and categorical variables as counts. We showed the distribution difference between the COVID-19 death and non-COVID-19 death according to cancer types by heat maps. Further, we used univariate and multivariate competing risk analyses to calculate the cumulative risk of COVID-19 death, adjusting for covariates such as age, gender, race, tumour summary stage, chemotherapy only, radiotherapy only, surgery only, combined chemotherapy and radiotherapy, combined chemotherapy and surgery, combined radiotherapy and surgery, combined chemotherapy, radiotherapy, and surgery, other therapy, and years after diagnosis. For multivariate analysis, we included variables with *P*-values <0.10 in univariate analysis in the multivariate regression model. We performed subgroup analysis simultaneously in different cancer types and age brackets. All statistical tests were two-sided, and we set the statistical significance at *P* < 0.05.

### Ethics statement

Ethics approval was not required for this study, which was based on the SEER database and was conducted in accordance with the Declaration of Helsinki.

## RESULTS

### Characteristics in the study population

Overall, 237 356 patients were enrolled in this study, with prostate cancer (13.7%), lung cancer (13.4%), breast cancer (11.4%), colon cancer (8.6%), bladder cancer (4.7%), non-Hodgkin lymphoma (NHL) (4.1%), and other cancer types constituted the remainder. There were more men than women (54.4% *vs* 45.6%), and the mean age in the study population was approximately 68.7 years. The white accounted for 80.3%, and 51.1% of patients died of cancer. The most common cancer type of COVID-19 death was CLL (11.0%). Chemotherapy (12.5%), radiotherapy (9.0%), and surgery (26.5%) were performed in patients respectively and often in combination (Table S1 in the **Online Supplementary Document**).

### COVID-19 death in cancer patients

**Figure 1:**
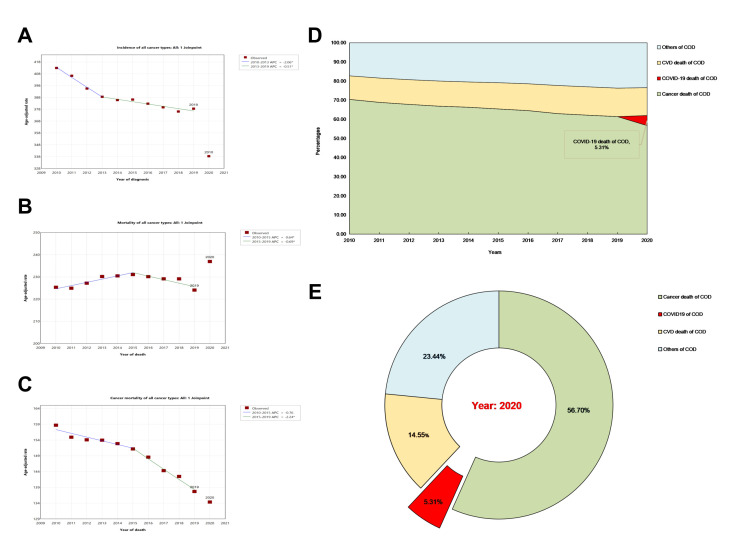
Age-adjusted incidence and mortality trends. **Panel A.** Decreasing trend in age-adjusted incidence of cancer between 2010–20. **Panel B.** Increasing trend in age-adjusted overall mortality of cancer in 2020. **Panel C.** Decreasing trend in age-adjusted cancer mortality between 2000–20. **Panel D.** Percentage of different causes of death between 2010–20. **Panel E.** Percentage of different causes of death in 2020, COVID-19 death accounted for 5.3%. Rates are per 100 000 and age-adjusted to the 2000 USA standard population (19 age groups-population census). Confidence intervals (Tiwari mod) are 95% for rates. The line segments of each curve were selected with the Joinpoint program, and the percentage associated with each line represents the annual percentage change during the indicated range of years. Asterisks indicate annual percentage changes that are significantly different from zero (*P* < 0.05). APC – annual percent change.

**Figure 2:**
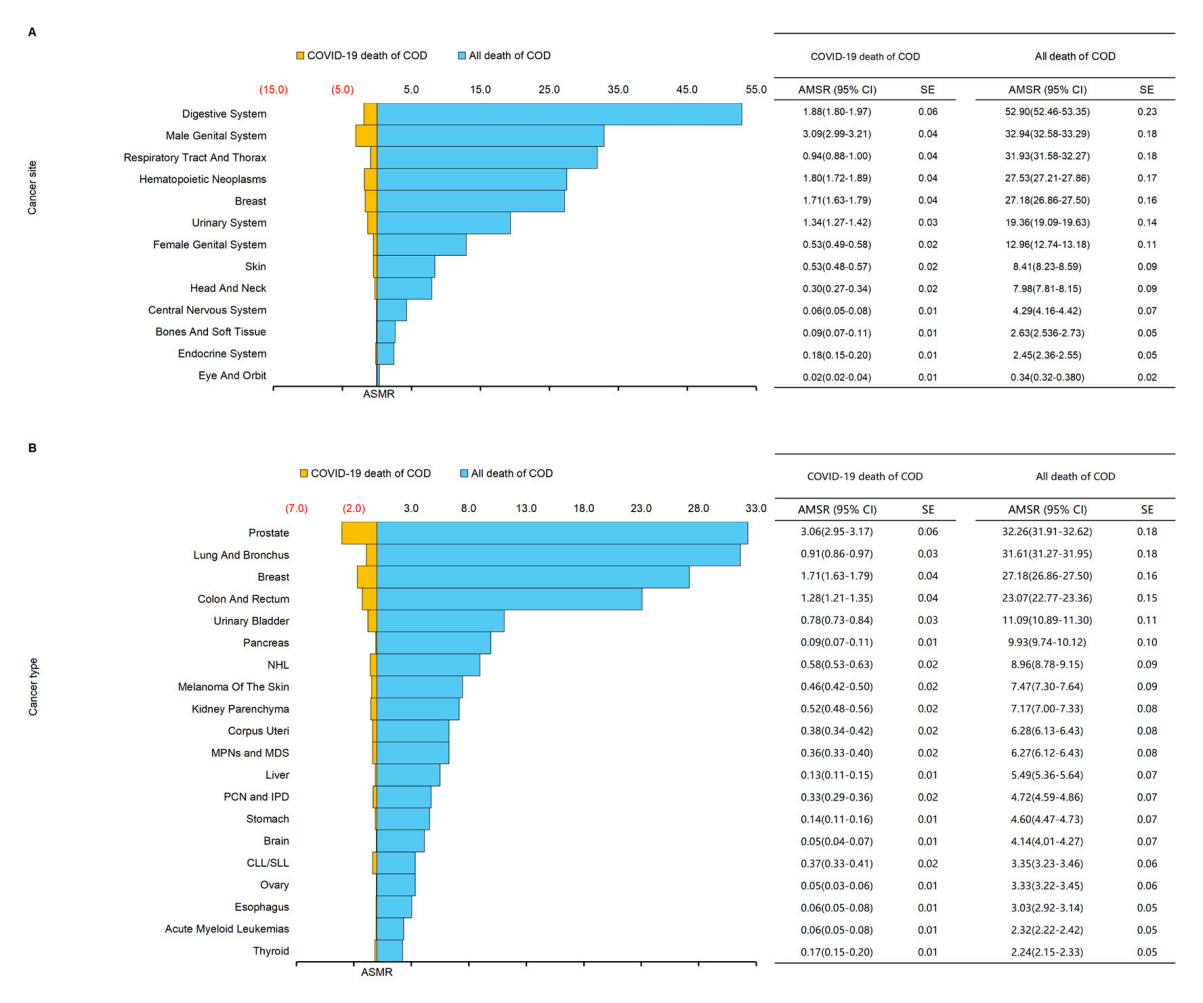
Different causes of death in patients with cancer in 2020. **Panel A.** All deaths and COVID-19 deaths are COD, according to cancer sites. **Panel B.** All deaths and COVID-19 deaths are COD according to cancer types. MPNs – myeloproliferative neoplasms, MDS – myelodysplastic syndromes, NHL – non-Hodgkin lymphomas, CLL – chronic lymphocytic leukaemia, SLL – small lymphocytic lymphoma, COD – cause of death, ASMR – age-standardised mortality rate.

Age-adjusted incidence gradually decreased (**Figure 1**, Panel A). It significantly decreased by 10% from 2019 (378 per 100 000 population) to 2020 (338 per 100 000 population). However, the mortality significantly increased (237 per 100 000 population) in 2020 (**Figure 1**, Panel B). This was accompanied by a continuous decline in cancer mortality (APC = –2.24) (**Figure 1**, Panel C). Considering the immune conditions in cancer patients and the COVID-19 pandemic in 2020, COVID-19 death may cause elevated overall mortality. 5.3% of cancer patients died of COVID-19 (**Figure 1**, Panel D–E). Analysing the overall mortality and COVID-19 mortality of cancer patients, we found that digestive system cancer was the most common, followed by male genital system cancer and respiratory tract and thorax cancer (**Figure 2**, Panel A). In contrast, the highest incidence of COVID-19 death was observed in male genital system cancer (3.09 per 100 000 population). Prostate cancer had the highest mortality of COVID-19 deaths (3.06 per 100 000 population) according to cancer types, ranking the first with an overall mortality of 32.26 per 100 000 population. The COVID-19 mortality of breast cancer was about 1.706 per 100 000 population, nearly twice as high as cancer in the lung and bronchus. Colon and rectum cancer had a COVID-19 mortality of 1.28/100 000. Although the overall mortality of lung and bronchus cancer was 31.61 per 100 000 population, the mortality of COVID-19 death was only 0.91 per 100 000 population (**Figure 2**, Panel B; Figure S2 in the **Online Supplementary Document****)**.

**Figure 3:**
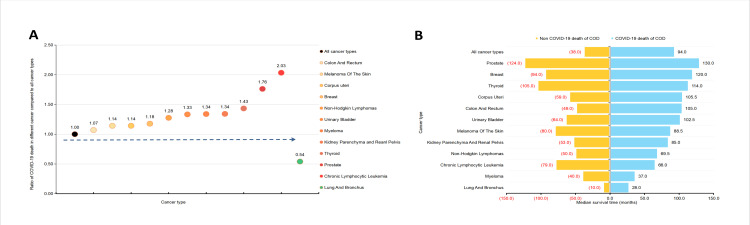
Survival analysis and distribution of COVID-19 death by cancer types. **Panel A.** Ratio of COVID-19 death in different cancer types compared to all cancer types. **Panel B.** Median survival time (COVID-19 death *vs* non-COVID-19 death) according to cancer types.

Considering that mortality is affected by incidence and total population, we calculated the proportion of COVID-19 deaths across various cancer types. CLL had the highest proportion of COVID-19 death (11.0%), followed by prostate cancer (9.5%), thyroid cancer (7.8%), kidney parenchyma and renal pelvis cancer (7.2%), myeloma (7.2%), urinary bladder cancer (7.2%), NHL (6.9%), breast cancer (6.3%), corpus uteri cancer (6.3%), colon and rectum cancer (5.8%), and melanoma of the skin (6.2%), while lung and bronchus cancer accounted for only 2.9% of COVID-19 deaths (Figure S3 in the **Online Supplementary Document**). Compared to COVID-19 deaths in the total population, the ratio of COVID-19 deaths in CLL patients reached 2.03. Prostate cancer had a ratio of 1.76, and lung and bronchus cancer was 46.0% lower than the total cancer population (**Figure 3**, Panel A).

### Survival analysis and distribution of COVID-19 death by cancer types

Based on the survival analysis of the total cancer population and the 12 specific cancer types, the median survival time of patients in the COVID-19 death group was significantly higher than that of non-COVID-19 death (94 *vs* 38 months) (**Figure 3**, Panel B; Figure S4 in the **Online Supplementary Document**). Kaplan-Meier curves and risk tables are presented in the Figure S5 in the **Online Supplementary Document**. Patients with prostate cancer, breast cancer, corpus uterine cancer, colon and rectum cancer, lung and bronchus cancer, kidney parenchyma and renal pelvis cancer, and NHL had significantly longer survival time compared to those who did not die of COVID-19. However, the result was completely opposite for patients with CLL (COVID-19 death *vs* non-COVID-19 death; 66 *vs* 79 months).

The results indicated that COVID-19 deaths gradually decreased with the year of cancer diagnosis. The heat maps showed great differences among different cancer types, with 52.3% of COVID-19 deaths occurring within one year from diagnosis in lung cancer patients and the highest number (28.9%) of NHL patients experiencing COVID-19 deaths within the same timeframe. Breast cancer had the highest number of COVID-19 deaths in one to five years from diagnosis (26.4%). The highest proportion (26.3%) of COVID-19 deaths in prostate cancer was 10–15 years from diagnosis. In addition, colon and rectum cancer (32.4%), urinary bladder cancer (28.9%), kidney parenchyma and renal pelvis cancer (28.8%), corpus uteri cancer (31.9%), melanoma of skin (29.0%) and myeloma (38.2%) were among the highest in one to five years from diagnosis. COVID-19 deaths occurred mainly five to 10 years from diagnosis in patients with thyroid cancer (25.8%) and CLL (30.1%). Most patients who died of COVID-19 infection were aged 60–80 years, with prostate cancer accounting for the highest proportion (78%). Men made up a larger proportion of patients with COVID-19 death in urinary bladder cancer, kidney cancer, melanoma of the skin, NHL, and CLL when excluding sex-specific cancer types. A greater proportion of women with colon and rectum cancer suffered from COVID-19 death. Notably, although males with lung cancer had slightly higher rates than females, the percentage of females who died of COVID-19 infection was higher than males (51.8% *vs* 48.2%). The cancer population with COVID-19 death was mainly in white individuals (Figure S6, Panel A in the **Online Supplementary Document**). The risk of COVID-19 death increased over time, with certain cancer types showing more pronounced trends. Prostate cancer, breast cancer, colon cancer, kidney cancer, and corpus uteri cancer exhibited an increasing risk of COVID-19 death with the increasing years of diagnosis (Figure S6, Panel B in the **Online Supplementary Document**). However, CLL showed an opposite trend. COVID-19 deaths were mainly observed in the age group of 60–80 years, while corpus uteri cancer patients were primarily in the 40–60 age group, and melanoma patients were predominantly aged >80 years. Men accounted for a higher proportion of most cancer types. Although most patients were white, COVID-19 deaths significantly diverged among black patients. Compared with the non-COVID-19 death group, differences concentrated in the localised cancer stage, in which kidney cancer was more than 24.4%, followed by uterine cancer (20.9%) and lung and bronchus cancer (18.1%).

### Risk factors for COVID-19 death

The COVID-19 death risk in other cancer types is shown in Figure S7 in the **Online Supplementary Document**. The multivariable analysis results showed that the risk of COVID-19 death remains higher in haematological malignancies such as Hodgkin Lymphoma (HL), NHL, CLL, and myeloma compared to prostate cancer. In the total cancer population, the risk of COVID-19 deaths increased with the increasing years from diagnosis (Figure S8–9 in the **Online Supplementary Document**). The highest risk in subgroups of patients with >15 years of cancer diagnosis (hazard ratio (HR) = 2.08; 95% confidence interval (CI) = 1.92–2.25, *P* < 0.001) was 0.5 fold more than the group with one to five years of cancer diagnosis (Figure S10, Panel A in the **Online Supplementary Document**). Patients aged 60–80 years (HR = 3.36; 95% CI = 1.65–6.85, *P* = 0.001) were susceptible to COVID-19 death. Males exhibited a higher COVID-19 death risk (HR = 1.14; 95% CI = 1.09–1.20, *P* < 0.001), while race, marital status, and treatments showed no significant impact. CLL (HR = 2.37; 95% CI = 2.07–2.71, *P* < 0.001), myeloma (HR = 1.83; 95% CI = 1.60–2.08, *P* < 0.001), NHL (HR = 1.27; 95% CI = 1.16–1.39, *P* < 0.001), and HL (HR = 1.54; 95% CI = 1.16–2.05, *P* = 0.003) were more likely to die of COVID-19 infection when prostate cancer as reference. In contrast, lung and bronchus cancer had a lower risk of COVID-19 death (HR = 0.61; 95% CI = 0.56–0.66, *P* < 0.001). The subgroup analysis results suggested the independent predictive role of years from cancer diagnosis for COVID-19 death (Figure S10, Panel B in the **Online Supplementary Document**). The risk of various cancer types were as follows: lung and bronchus cancer HR = 1.29 (95% CI = 1.20–1.40, *P* < 0.001), colon and rectum cancer HR = 1.22 (95% CI = 1.16–1.29, *P* < 0.001), urinary bladder HR = 1.22 (95% CI = 1.15–1.30, *P* < 0.001), NHL HR = 1.17 (95% CI = 1.11–1.23, *P* < 0.001), kidney cancer HR = 1.15 (95% CI = 1.04–1.24, *P* < 0.001), breast cancer HR = 1.11 (95% CI = 1.06–1.16, *P* < 0.001), and corpus uteri cancer HR = 1.10 (95% CI = 1.01–1.19, *P* = 0.03). The opposite result was obtained in CLL (HR = 0.81; 95% CI = 0.73–0.90, *P* < 0.001). We found profound differences between hematologic malignancies and solid tumours. Finally, we obtained consistent findings through subgroup analyses by age (Figure S10, Panel C in the **Online Supplementary Document****)**.

## DISCUSSION

Our study findings demonstrate the burden and risk factors of COVID-19 death for different cancer types, including nearly 240 000 patients. The results support the feasibility and safety of continuing cancer treatment during SARS-CoV-2 infection, particularly for long-term cancer survivors, who should receive ongoing community support for preventive measures.

Our findings demonstrated that COVID-19 death caused approximately 5.3% of all deaths in cancer patients, which is third to cancer death and cardiovascular diseases. The overall mortality for cancer patients has increased, which may be due to COVID-19 death. Between 2020–22, the global COVID-19 mortality ranged from 0.1–1% [1,24]. Despite dynamic changes in mortality, COVID-19 still has a significant impact on cancer patients. A previous study reported that COVID-19 infection accounted for two-thirds of the underlying causes of death [25]. Furthermore, the COVID-19 pandemic posed a decrease in cancer incidence of nearly 10% [26] due to disruptions in health care services and delays in cancer screening, diagnosis, and treatment [24,25,27,28]. Low vaccination rate, compromised immune system, and fear of infection may lead to decreased screenings for cancer, potentially resulting in missed diagnoses and delayed treatments [29–31]. These factors could result in more deaths from COVID-19 in cancer patients. Therefore, it is crucial to pay attention to enhancing awareness of COVID-19 infection in cancer patients.

Previous studies have reported an independent association between cancer types and COVID-19 mortality in hospitalised patients [12,32]. Our results suggest that CLL and prostate cancer have higher mortality. Interestingly, the mortality of respiratory system cancer is low. It is consistent with a previous study that found patients with lung cancer were not at high risk of severe events compared to other cancer types [5]. Conversely, previous research has shown that SARS-CoV-2 primarily affected lungs, and COVID-19 mortality was significantly higher in patients with lung cancer [33]. This finding prompts our consideration that lung cancer patients may have a higher risk of COVID-19 infection but a lower risk of mortality, and further research is needed. Another cancer type that has received significant attention is CLL, which accounted for the highest proportion of COVID-19 deaths. Consistent with our results, Yang et al. suggest that patients with haematological malignancies experience severe forms of COVID-19 compared to those with solid tumours [34]. This may be due to the immunosuppression caused by the primary cancer in the blood. In addition, patients with CLL who suffer from COVID-19 have poor survival time, probably due to lymphocyte depletion and hypoalbuminemia [7,20,35,36], leading to higher rates of acute respiratory distress syndrome, thrombotic complications, and in-hospital mortality [37,38]. Furthermore, patients with haematological malignancies were more susceptible to the effects of SARS-CoV-2 mutations [39]. The survival data of CLL is completely consistent with the highest proportion of COVID-19 deaths. It is imperative to implement additional COVID-19 preventive measures in CLL patients.

Moreover, prostate cancer has the highest mortality of COVID-19 death according to cancer type, while breast cancer also has a higher mortality. Prostate cancer and breast cancer are the leading cancers in males and females, respectively [25], and often undergo surgical treatment. However, surgery may be postponed due to COVID-19 infection. The initiation rate of adjuvant systemic therapy for early-stage breast cancer (tumour stage one/two) has decreased by 25% [40]. Interruption or postponement of treatment exacerbates the cancer progression and increases the risk of COVID-19 death, which affects overall mortality [41]. It is especially important to assess the risk of COVID-19 death in patients and designate treatment plans. Lastly, attention should be paid to cancers of the urinary system (kidney and urinary bladder), as COVID-19 mortality is higher in urinary system cancer than the average of all cancer types. Viral infection further worsens the condition in the urinary system. About 25.1% of patients may experience acute kidney injury [42]. Clinical manifestations of COVID-19 infection range from mild proteinuria to progressive acute kidney injury and even the need for renal replacement therapy [43,44]. Previous studies suggested that the kidney should be considered a high-risk organ using single-cell sequence and imaging findings [45–47]. These results suggest a higher risk of COVID-19 death in kidney and urinary bladder cancers. Variations in angiotensin-converting enzyme 2 (ACE2), transmembrane protease serine 2, A disintegrin and metalloprotease 17 (ADAM17), and Dedicator of cytokinesis 2 (DOCK2) levels, influenced by cancer types, differ among populations [48–50]. These differences may explain disparities in viral entry mechanisms and host immune responses. In conclusion, it is crucial to consider the specific risks and implications of COVID-19 infection in different cancer types and implement appropriate preventive measures and treatment strategies.

In this study, we determined the relationship between COVID-19 death and demographic factors, treatments, and years from cancer diagnosis. We revealed that the proportion of COVID-19 deaths was highest among white patients, while the disparity between the proportion of COVID-19 cases and non-COVID-19 deaths was more pronounced among black patients. Previous studies have also highlighted racial disparities in COVID-19 infection [51–53]. Black patients with more comorbidities experienced significantly more severe COVID-19 and had worse outcomes due to a low likelihood of receiving anti-COVID-19 treatment [54–56]. Disparities in COVID-19 mortality among racial groups result from biological and social determinants of health. Biologically, genetic variations in immune response pathways may influence susceptibility to severe outcomes, with significant differences in inflammatory markers and gene expression patterns in COVID-19-related pathways, such as interleukin-1 beta and genes like glutathione S-transferase M1, C-C motif chemokine ligand 3 like 3, and coagulation factor VIII associated 2 [57,58]. Non-white patients have higher 30-day mortality rates, and race significantly affects prognosis [59]. Socio-economic inequalities exacerbate these disparities through limited health care access, poor living conditions, and higher occupational exposure risks [57,60,61]. In conclusion, racial disparities in COVID-19 outcomes may indicate differences in access to health care services, as well as a potential correlation between race and infection. Future research should focus on the mechanisms exacerbating health disparities during the COVID-19 infection, and provide more targeted health care policies.

Additionally, we found a nonlinear relationship between age and COVID-19 mortality. Cancer survivors aged 60–80 years have the highest risk of COVID-19 death, surpassing the risk of those aged >80 years. It is known that cancer typically occurs in older populations, often with comorbidities that may increase the risk of adverse outcomes. However, further research is needed to understand this phenomenon in patients aged 60–80 years. Similar to previous studies, male is a risk factor for COVID-19 death. These findings help identify cancer patients at higher risk of COVID-19 death.

Notably, we found that previous cancer treatments were not associated with a higher risk of COVID-19 death. Some reports support our findings show that past chemotherapy or early treatment and delayed treatment does not significantly affect the mortality of COVID-19 [56,62]. Similar observations have been made in immunotherapy, hormonal therapy, targeted therapy, and radiation therapy [35,37,63]. This finding prompts us to consider whether effective anticancer treatments should continue to be provided and guide decision-making during the SARS-CoV-2 infection. In addition to our efforts to prevent COVID-19 infection and reduce COVID-19 mortality, we should also pay attention to anticancer treatment and master the optimal treatment time.

Of particular concern is the impact of years from diagnosis on COVID-19 death, for which there are currently no relevant studies or reports. One possible explanation for this finding may be the neglect of prophylaxis for patients with longer survival time or decreased perception of the risk of COVID-19 infection. Previous studies have shown differences in the fear of COVID-19 infection risk between patients with longer diagnosis times (>12 months) [64]. An increase in comorbidity prevalence and ageing may contribute to the higher risk of death from COVID-19 in cancer patients with longer diagnosis years [65–68]. These findings reveal that active anti-cancer treatment plans should be developed for early-stage cancer patients, while attention should be paid to prevent viral infection in late-stage cancer patients. For haematological malignancies, especially CLL, better prevention and treatments are needed at the beginning of diagnosis due to the high mortality and short survival time of CLL. This highlights the importance of further investigating clinical factors and population characteristics associated with COVID-19 death in cancer patients.

### Findings

Our research is the first large-scale study on COVID-19 death in cancer patients. It includes information on cancer patients registered at the National Cancer Database in 17 states, the highest mortality of COVID-19 death was in patients with chronic lymphocytic leukaemia (11.0%), followed by prostate cancer (9.5%). Interestingly, lower risk was found in patients with lung and bronchus cancer (2.9%). Remarkably, we reveal that receiving radiation therapy, chemotherapy, and surgical treatment did not show a significant correlation with COVID-19 death. Crucially, we found that years from diagnosis independently predict COVID-19 death in cancer patients. This study provides evidence for timely treatment of recently diagnosed patients and attention to infection prevention in long-term survivors, facilitating more prompt risk assessment and treatment for various types of cancer.

### Limitations

The virus continues to mutate, and immune responses gradually decline; patients can experience recurrent infections with the novel coronavirus. Until now, vaccination remains an effective measure for preventing COVID-19. Unfortunately, our research data lacks information on patients who have received the COVID-19 vaccine, as well as laboratory indicators to assess the immune system after COVID-19 infection. Additionally, this study lacks data on anti-virus drugs used in cancer patients, preventing us from understanding the relationship between antiviral medications and cancer treatment. Furthermore, this study suggests lower COVID-19 mortality among lung cancer patients, which may be influenced by regional differences and potential confounders. Therefore, further exploration of the underlying mechanisms is needed. Lastly, the database did not provide information on comorbidities. However, we have included relevant diseases as competing events in the cause-of-death analysis to minimise the influence of confounding factors on the results. Nevertheless, this study provides evidence for timely treatment of recently diagnosed patients and attention to infection prevention in long-term survivors, facilitating more prompt risk assessment and treatment for various types of cancer.

## CONCLUSIONS

The burden of COVID-19 death is different across cancer types. COVID-19 mortality was highest in CLL and prostate cancer, while patients with lung and bronchus cancer had a lower risk in this study. Years from diagnosis independently predict COVID-19 death. In this study, we provided evidence for timely treatment of recently diagnosed patients and attention to infection prevention in long-term survivors, facilitating more prompt risk assessment and treatment for various types of cancer.

## Additional material


Online Supplementary Document
