# Supplementary material for: The burden of COVID-19 death for different cancer types: a large population-based study
Source: J Glob Health. 2025 Feb 14;15:04046. doi: 10.7189/jogh.15.04046 (PMC11825124; doi:10.7189/jogh.15.04046)

Supplement to: Mo Y, Wei D, Chen X, Zhang Z, Huo W, Wu M, Chen D, Yu J. The burden of COVID-19 death for different cancer types: a large population-based study. J Glob Health. 2025;15:04046.

Online Supplementary Document

Table S1. Patient Characteristics.
Fig. S1. Patient data selection process. SEER=Sureillance, Epidemiology, and End Results.
Fig. S2. All death and COVID-19 death as COD according to cancer types. COD, Cause of Death. ASMR, Age-Standardized Mortality Rate.
Fig. S3. Percentage of COVID-19 death in different cancer types.
Fig. S4. Kaplan-Meier estimations of survival time comparing COVID-19 and Non-OVID-19 in all cancer types.
Fig. S5. Kaplan-Meier estimations of survival time comparing COVID-19 and Non COVID-19 in different cancer types.
Fig. S6. Characteristic distribution of cancer patients who died of COVID-19. **Panel A.** Basic characteristic distribution of cancer patients who died of COVID-19. **Panel B.** Percentage of COVID-19 death compared to Non-COVID-19 death.
Fig. S7. The risk of COVID-19 death in different cancer types.
Fig. S8. Hazard ratio of COVID-19 death compared to Non-COVID-19 death in total cancer population. Years from cancer diagnosis was an independent predictive factor for COVID-19 death.
Fig. S9. Cumulative incidence of COVID-19 death according to years from diagnosis.
Fig. S10. Years from diagnosis was an independent predictive factor for COVID-19 death. **Panel A.** Hazardous ratio of years from diagnosis in all cancer types. **Panel B.** Hazardous ratio of years from diagnosis in different types of cancers. **Panel C.** Hazardous ratio of years from diagnosis in age subgroups.

Supplementary Figure S1

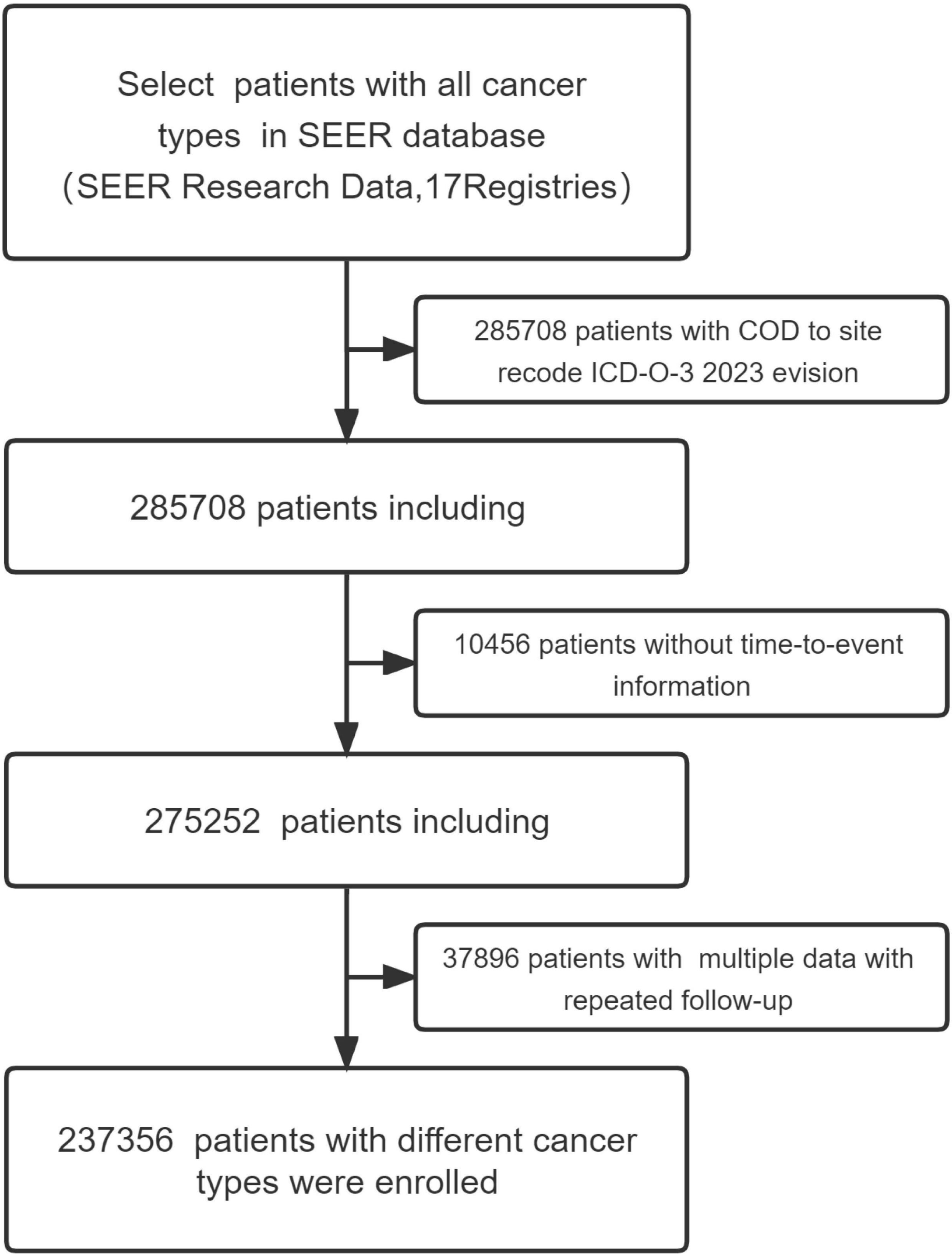

Supplementary Figure S2

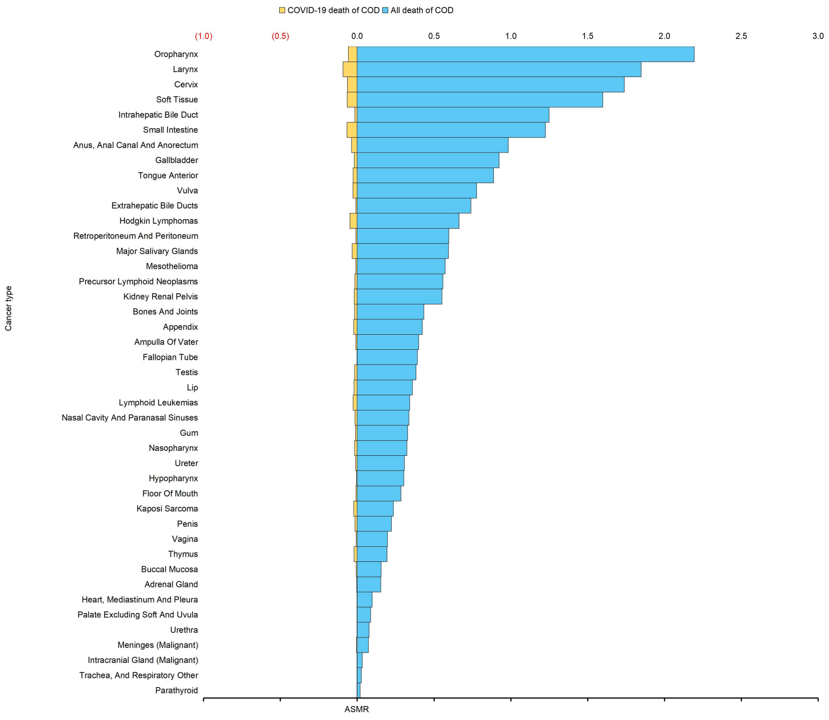

COVID-19 death of COD		All death of COD	
AMSR (95% CI)	SE	AMSR (95% CI)	SE
0.06(0.04-0.07)	0.01	2.19(2.11-2.29)	0.05
0.09(0.07-0.11)	0.01	1.85(1.77-1.93)	0.04
0.04(0.05-0.08)	0.01	1.74(1.66-1.83)	0.04
0.07(0.05-0.08)	0.01	1.60(1.52-1.68)	0.04
0.02(0.01-0.02)	0.00	1.25(1.18-1.32)	0.04
0.07(0.05-0.08)	0.01	1.23(1.16-1.30)	0.04
0.04(0.03-0.05)	0.01	0.980.92-1.05)	0.03
0.04(0.01-0.03)	0.00	0.92(0.87-0.99)	0.03
0.03(0.02-0.04)	0.01	0.89(0.83-0.95)	0.03
0.03(0.02-0.04)	0.01	0.78(0.72-0.83)	0.03
0.01(0.00-0.02)	0.00	0.74(0.69-0.80)	0.03
0.05(0.04-0.06)	0.01	0.66(0.61-0.72)	0.03
0.01(0.00-0.02)	0.00	0.60(0.55-0.65)	0.02
0.03(0.02-0.05)	0.01	0.59(0.55-0.64)	0.02
0.01(0.00-0.02)	0.00	0.57(0.53-0.62)	0.02
0.02(0.01-0.03)	0.00	0.56(0.51-0.61)	0.03
0.02(0.01-0.03)	0.00	0.55(0.51-0.60)	0.02
0.02(0.01-0.03)	0.00	0.43(0.39-0.48)	0.02
0.02(0.02-0.04)	0.01	0.42(0.39-0.47)	0.02
0.01(0.00-0.02)	0.00	0.40(0.36-0.44)	0.02
0.00(0.00-0.01)	0.00	0.39(0.35-0.43)	0.02
0.02(0.01-0.03)	0.00	0.38(0.34-0.43)	0.02
0.02(0.01-0.03)	0.00	0.36(0.32-0.40)	0.02
0.03(0.02-0.04)	0.01	0.34(0.31-0.38)	0.02
0.01(0.01-0.02)	0.00	0.34(0.30-0.38)	0.02
0.01(0.01-0.02)	0.00	0.33(0.29-0.37)	0.02
0.02(0.01-0.03)	0.00	0.32(0.29-0.36)	0.02
0.01(0.01-0.02)	0.00	0.31(0.27-0.34)	0.02
0.01(0.00-0.01)	0.00	0.30(0.27-0.34)	0.02
0.01(0.00-0.02)	0.00	0.29(0.25-0.32)	0.02
0.02(0.02-0.04)	0.01	0.24(0.20-0.27)	0.02
0.01(0.01-0.02)	0.00	0.22(0.30-0.26)	0.02
0.01(0.00-0.02)	0.00	0.20(0.17-0.23)	0.01
0.02(0.01-0.03)	0.01	0.19(0.17-0.22)	0.01
0.01(0.00-0.02)	0.00	0.16(0.13-0.18)	0.01
0.00(0.00-0.01)	0.00	0.15(0.13-0.18)	0.01
0.00(0.00-0.01)	0.00	0.10(0.08-0.12)	0.01
0.00(0.00-0.01)	0.00	0.09(0.07-0.11)	0.01
0.00(0.00-0.01)	0.00	0.08(0.06-0.10)	0.01
0.01(0.03-0.01)	0.00	0.07(0.06-0.09)	0.01
0.00(0.00-0.01)	0.00	0.03(0.02-0.05)	0.01
0.00(0.00-0.01)	0.00	0.03(0.02-0.04)	0.01
0.00(0.00-0.01)	0.00	0.02(0.01-0.03)	0.01

Supplementary Figure S3

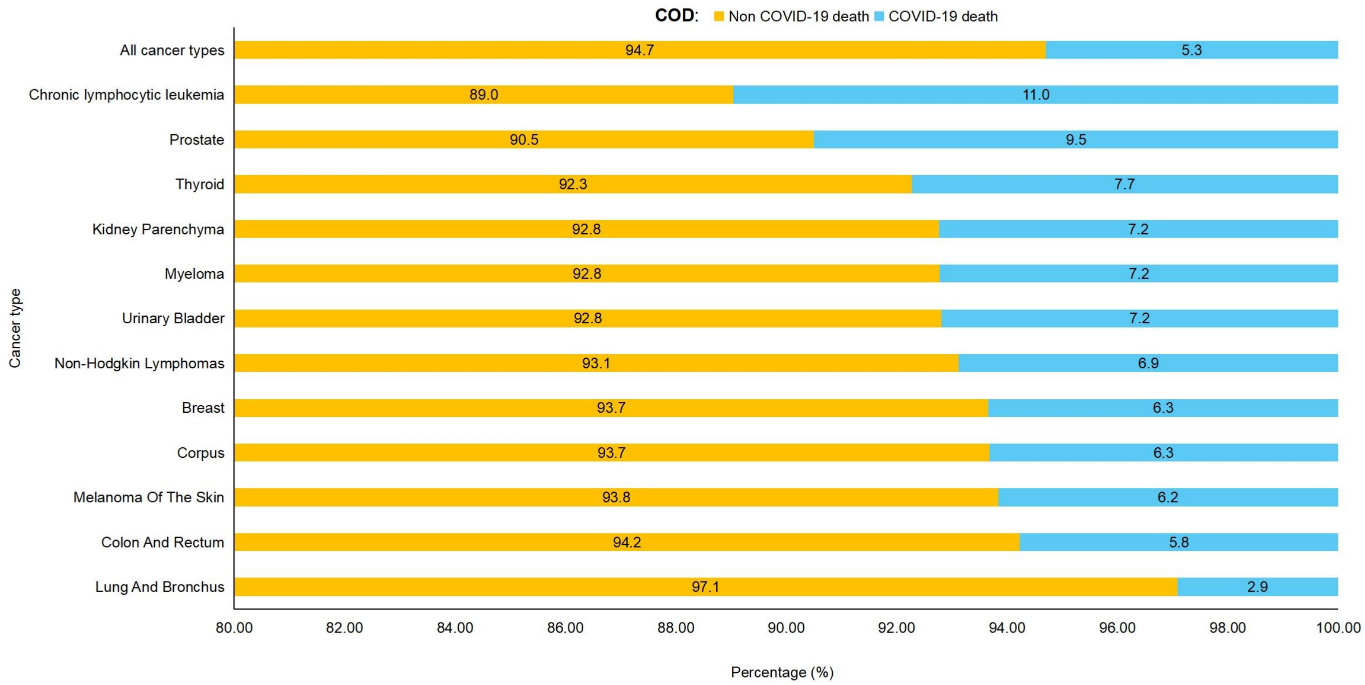

Supplementary Figure S4

Survival by all cancer types

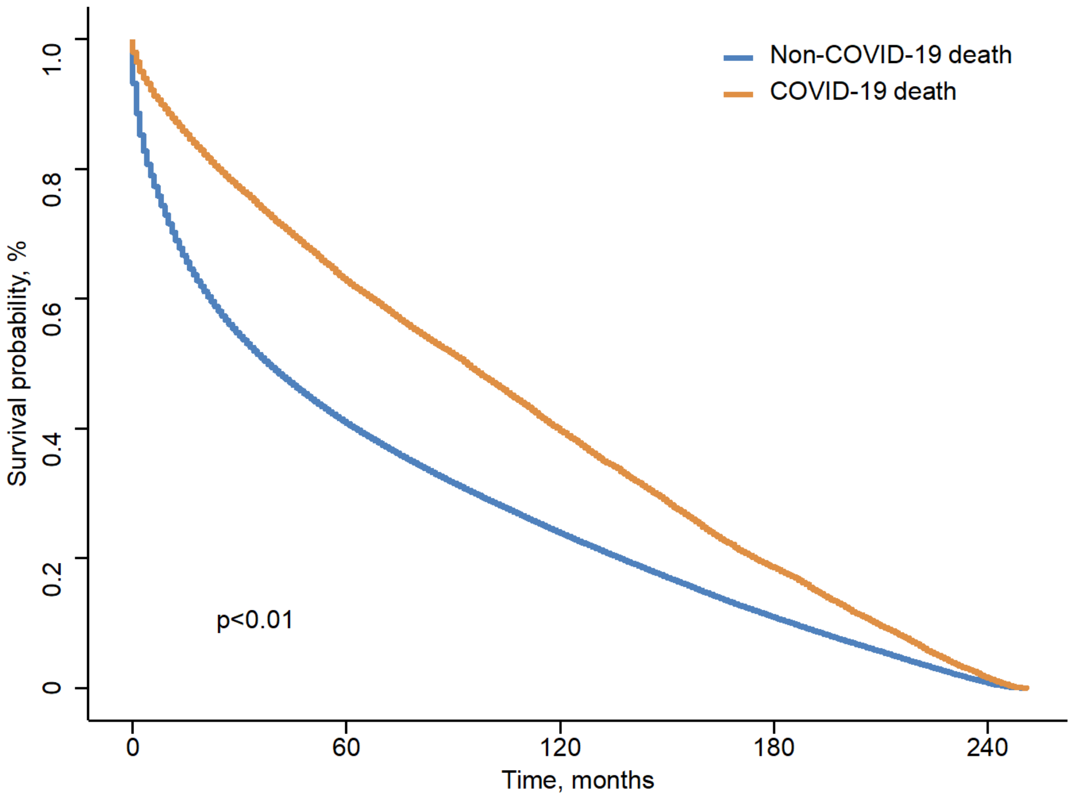

Number at risk

Non-COVID-19 death	224559	92327	54113	24796	2015
COVID-19 death	12797	8078	5125	2398	223

Supplementary Figure S5

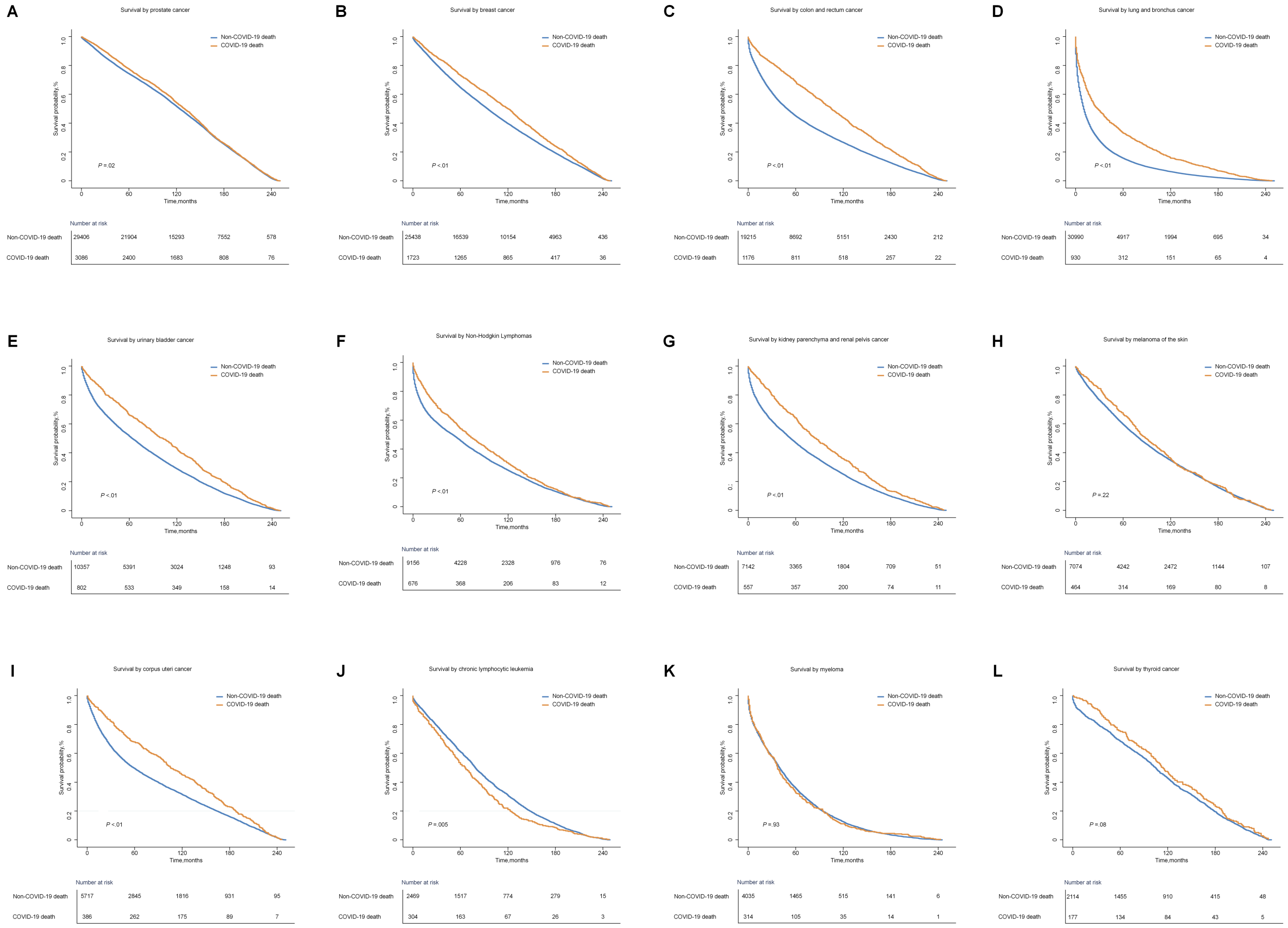

Supplementary Figure S6

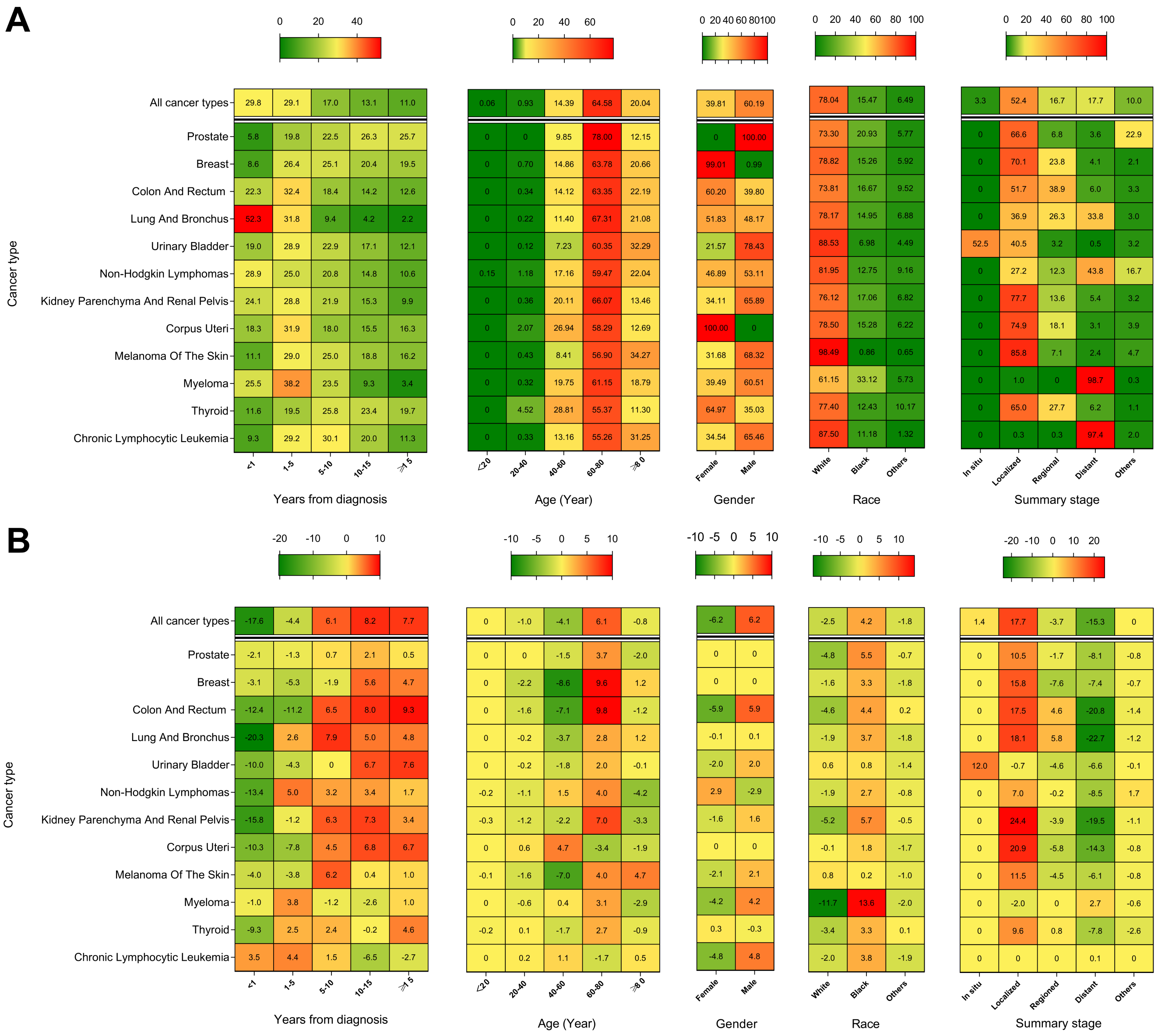

Supplementary Figure S7

Variables	Non-COVID-19 death	COVID-19 death	HR (95%CI)	p value
**Cancer type**				
Stomach	4410	136	0.59(0.49-0.71)	<0.001
Liver	5476	140	0.42(0.36-0.51)	<0.001
Larynx	1819	95	0.67(0.54-0.82)	<0.001
Pancreas	9879	95	0.24(0.19-0.29)	<0.001
Small Intestine	1165	67	0.83(0.65-1.06)	0.14
Soft Tissue	1508	68	0.65(0.51-0.83)	<0.001
Cervix	1601	67	0.83(0.65-1.07)	0.15
Esophagus	3073	63	0.45(0.35-0.58)	< 0.001
Acute Myeloid Leukemias	2170	60	0.88(0.67-1.15)	0.35
Oropharynx	301	11	0.74(0.41-1.35)	0.32
Brain	3988	55	0.26(0.20-0.35)	<0.001
Hodgkin Lymphomas	588	48	1.54(1.16-2.05)	0.003
Ovary	3263	45	0.32(0.24-0.43)	<0.001
Anus, Anal Canal And Anorectum	989	38	0.65(0.47-0.90)	0.009
Major Salivary Glands	568	33	0.79(0.56-1.12)	0.19
Vulva	764	29	0.49(0.34-0.70)	<0.001
Tongue Anterior	1782	53	0.45(0.34-0.60)	<0.001
Lymphoid Leukemias	465	12	0.98(0.55-1.76)	0.95
Appendix	409	23	0.96(0.63-1.45)	0.84
Kaposi Sarcoma	189	23	1.61(1.07-2.43)	0.02
Thymus	363	27	1.32(0.90-1.94)	0.16
Lip	348	19	0.48(0.31-0.76)	0.001
Gallbladder	927	19	0.46(0.29-0.73)	<0.001
Nasopharynx	320	17	1.09(0.68-1.75)	0.73
Bones And Joints	374	18	0.88(0.55-1.41)	0.58
Testis	333	17	0.76(0.47-1.23)	0.26
Intrahepatic Bile Duct	1255	15	0.27(0.16-0.46)	< 0.001
Nasal Cavity And Paranasal Sinuses	356	13	0.59(0.34-1.02)	0.06
Penis	213	15	0.76(0.46-1.27)	0.30
Gum	798	29	0.52(0.36-0.74)	< 0.001
Ureter	297	11	0.49(0.27-0.88)	0.02
Trachea, And Respiratory Other	72	3	0.79(0.26-2.43)	0.68
Others	21383	838	0.77(0.70-0.84)	<0.001

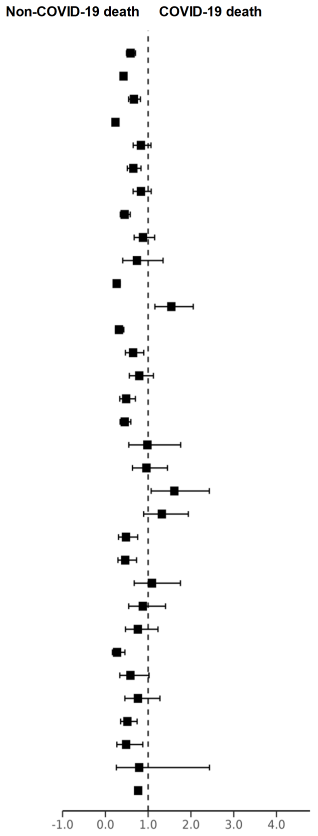

Supplementary Figure S8

Variables	Non COVID-19 death	COVID-19 death	HR (95%CI)	p value
**Age(years)**			1.15(1.12-1.18)	<0.001
<20	704	8		
20-40	4264	119	1.92(0.93-3.98)	0.08
40-60	41469	1842	2.68(1.32-5.46)	0.01
60-80	131419	8264	3.36(1.65-6.85)	0.001
≥80	46703	2564	3.32(1.63-6.77)	0.001
**Gender**			1.22(1.17-1.27)	<0.001
Female	103195	5095		
Male	121364	7702	1.14(1.09-1.20)	<0.001
**Summary Stage**			0.83(0.82-0.85)	<0.001
In situ	4197	421		
Localized	77927	6702	0.78(0.68-0.90)	0.001
Regional	45729	2131	0.60(0.52-0.69)	<0.001
Distant	74158	2261	0.39(0.34-0.46)	<0.001
Others	22548	1282	0.54(0.46-0.63)	<0.001
**Cancer type**			0.99(0.98-0.99)	<0.001
Prostate	29406	3086		
Breast	25438	1723	0.83(0.76-0.90)	<0.001
Colon And Rectum	19215	1176	0.80(0.74-0.87)	<0.001
Lung And Bronchus	30990	930	0.61(0.56-0.66)	<0.001
Urinary Bladder	10357	802	0.66(0.59-0.74)	<0.001
Non-Hodgkin Lymphomas	9156	676	1.27(1.16-1.39)	<0.001
Kidney Parenchyma and Renal Pelvis	7142	557	0.89(0.81-0.98)	0.02
Melanoma Of The Skin	7074	464	0.62(0.55-0.68)	<0.001
Corpus Uteri	5717	386	0.85(0.75-0.95)	0.01
CLL	2469	304	2.37(2.07-2.71)	<0.001
Myeloma	4035	314	1.83(1.60-2.08)	<0.001
Thyroid	2114	177	0.93(0.80-1.09)	0.38
**Chemotherapy only**			0.84(0.38-1.88)	0.67
No/Unknown	195736	11841		
Received	28823	956	0.84(0.38-1.90)	0.68
**Radiotherapy only**			1.03(0.46-2.31)	0.94
No/Unknown	204882	11222		
Received	19677	1575	0.94(0.42-2.12)	0.88
**Surgery only**			1.03(0.46-2.31)	0.94
No/Unknown	166662	7871		
Received	57897	4926	1.05(0.47-2.37)	0.90
**Radiotherapy and Chemotherapy**			0.62(0.28-1.39)	0.25
No/Unknown	208226	12361		
Received	16333	436	0.68(0.30-1.54)	0.35
**Radiotherapy and Surgery**			0.97(0.44-2.17)	0.94
No/Unknown	213112	11922		
Received	11447	875	0.94(0.42-2.10)	0.87
**Chemotherapy and Surgery**			0.78(0.35-1.75)	0.54
No/Unknown	207291	12028		
Received	17268	769	0.84(0.28-1.45)	0.28
**Chemotherapy,Surgery and Radiotherapy**			0.59(0.26-1.33)	0.20
No/Unknown	211363	12347		
Received	13196	450	0.64(0.28-1.45)	0.28
**No therapy**			0.98(0.44-2.20)	0.96
No/Unknown	164480	9981		
Received	60079	2816	0.92(0.41-2.08)	0.85
**Years from diagnosis**			1.28(1.27-1.30)	<0.001
<1	66847	1560		
1-5	65385	3159	1.55(1.45-1.66)	<0.001
5-10	38214	2953	1.79(1.66-1.93)	<0.001
10-15	29317	2727	1.90(1.76-2.06)	<0.001
≥15	24796	2398	2.08(1.92-2.25)	<0.001

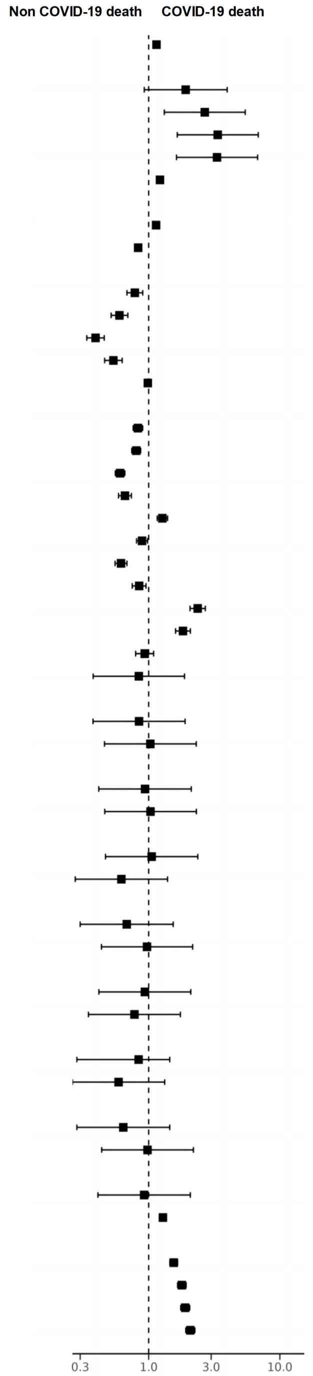

Supplementary Figure S9

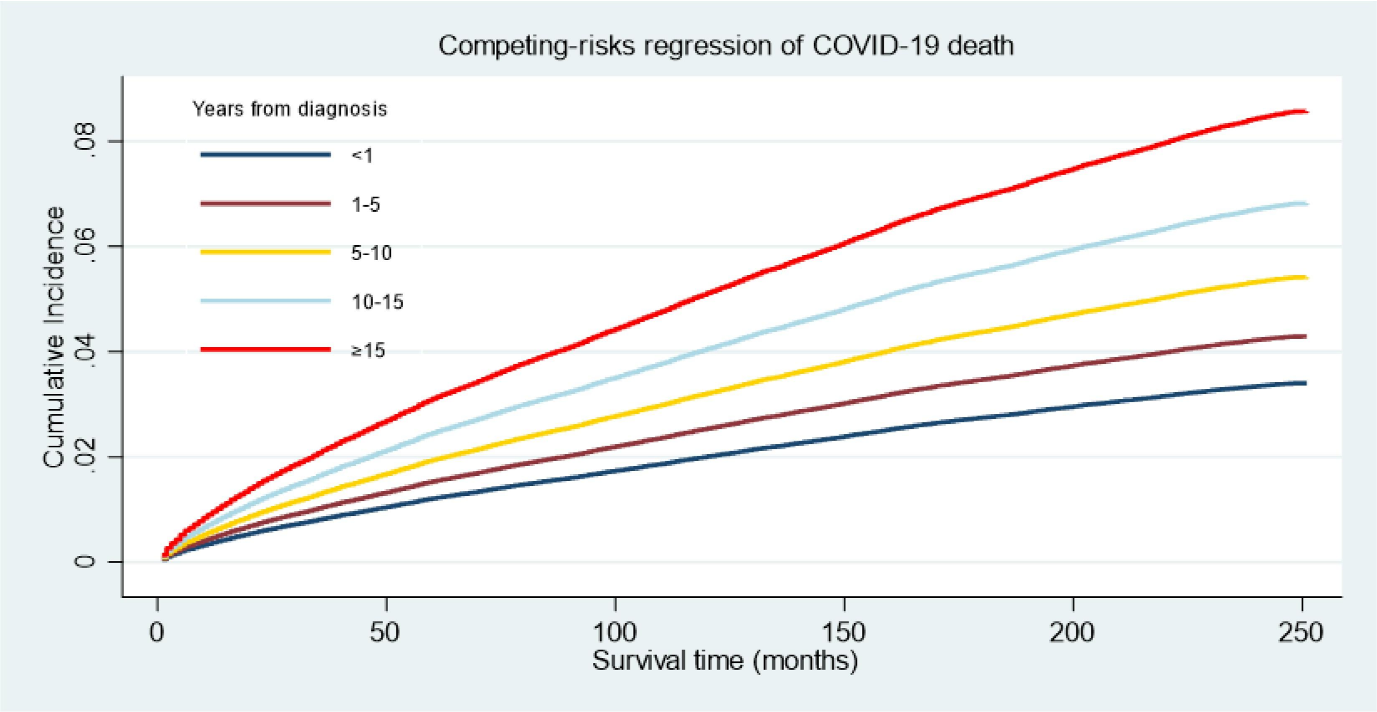

Supplementary Figure S10

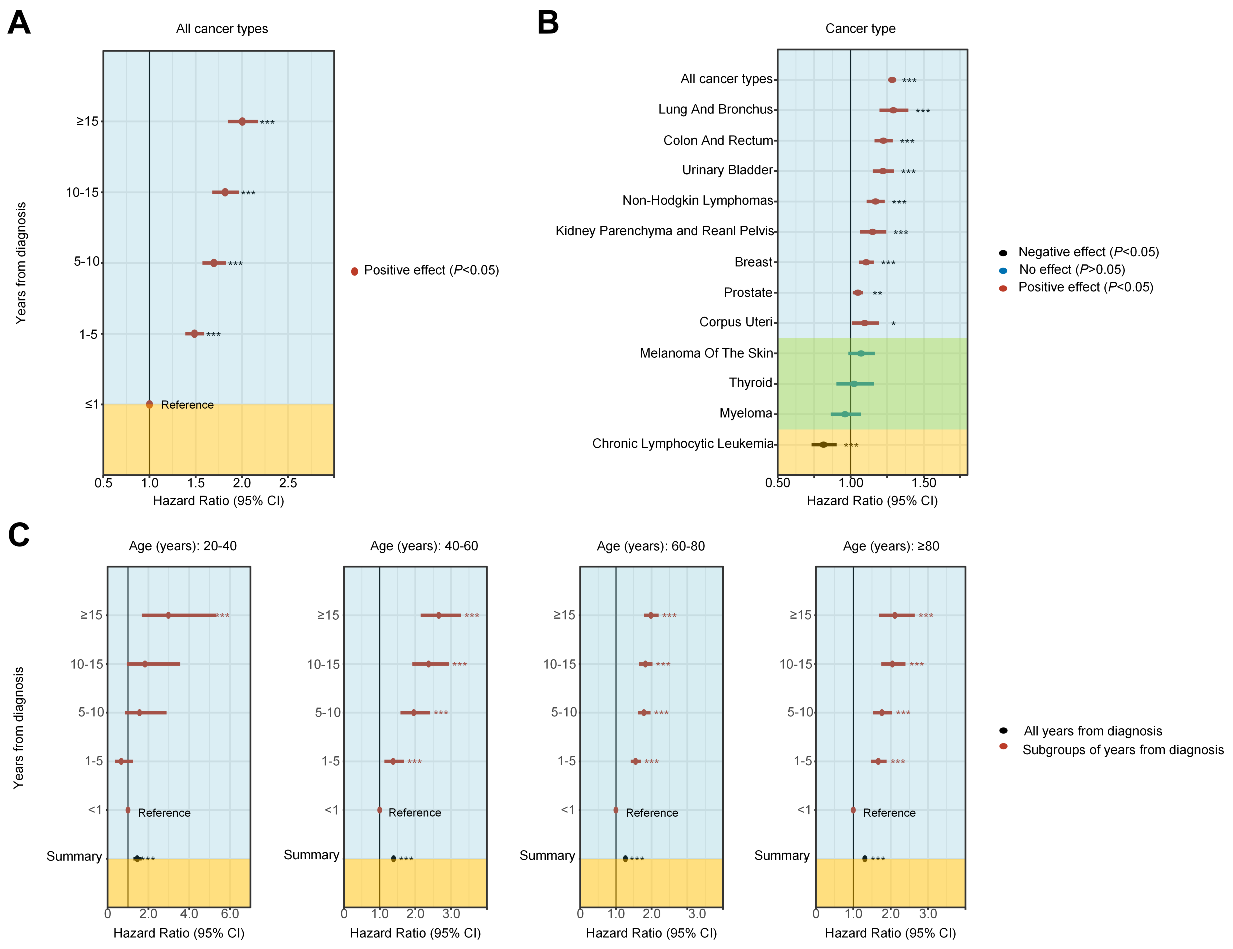

Supplement: Online Supplementary Document [file jogh-15-04046-s001.pdf]
